# Forearm Compartment Syndrome Caused by Reperfusion Injury

**DOI:** 10.1155/2014/931410

**Published:** 2014-07-10

**Authors:** Ufuk Sayar, Tanıl Özer, İlker Mataracı

**Affiliations:** Cardiovascular Surgery Department, Ahi Evren Thoracic and Cardiovascular Surgery Education and Research Hospital, 61040 Trabzon, Turkey

## Abstract

Compartment syndrome is commonly seen following lower extremity ischemia. However, upper extremities' compartment syndrome, especially after any vascular surgical procedures, is infrequent. Herein we report a case of an acute forearm compartment syndrome that was developed after delayed brachial artery embolectomy.

## 1. Introduction

Compartment syndrome is a clinical condition that is characterized by functional loss of muscle and nerve tissues and develops as a result of ischemia which can occur due to increased perfusion pressure within closed muscle fascia of the extremities. Tissue reperfusion after ischemia can cause reperfusion syndrome. Reperfusion syndrome includes both local (compartment syndrome) and systemic (acidosis, hypercalcemia, renal, hepatic, intestinal and pulmonary insufficiency, arrhythmia, and cardiac arrest) adverse effects [1]. Nearly one-fifth of acute extremity ischemia is related to upper extremity. Embolism is associated with up to 90% of cases and 75% are originated from the heart. Following successful brachial embolectomy, 95% to 98% of patients survive symptom free whereas 2% of the patients suffer from long-term arm claudication [2]. Herein, we reported a patient who ran into acute forearm compartment syndrome after brachial embolectomy due to left upper extremity embolism.

## 2. Case Presentation

An 81-year-old woman was referred to our clinic with complaints of sudden onset left arm pain; cooling and tenderness started 15 days previously. On physical examination, her left arm was cool and pale without any neurological deficit. Left axillary pulse was palpable whereas brachial and distal pulses (radial and ulnar) were absent. Right arm blood pressure was measured 170/90 mmHg. Atrial fibrillation was evident in electrocardiography. An urgent surgery was performed with local anesthesia. The left brachial artery was exposed and 3F embolectomy catheter (Fogarty Edwards Lifesciences, USA) was moved forward to axillary, radial, and ulnar arteries, consecutively. Fresh and mature thrombus was extracted. Radial and ulnar pulses became slightly felt. On follow-up, the patient complained of a severe pain (resistant to painkillers) and numbness of upper left extremity. Edema, stiffness, and pallor developed and the pulses of left forearm vanished in an hour. Urgent fasciotomy incisions were implemented to volar and dorsal faces of forearm as a rapid compartment syndrome occurred. The symptoms were improved after the implementation. But radial and ulnar pulses were still absent. The perfusion of hand was evaluated by noninvasive pulse oximeter which showed %95 oxygen saturation (SaO_2_). The fasciotomy wounds were treated with nonadherent dressing. In the postoperative follow-up, acute renal failure developed. Hematocrit (HCT): 29.7, white blood cell (WBC): 9000, urea: 116 mg/dL, and creatinine: 3.0 mg/dL were measured in the postoperative first day. This patient's laboratory results showed an acute renal failure. With hydration and forced diuresis treatment, renal failure was improved (HCT: 28.2, WBC: 9000, urea: 25 mg/dL, and creatinine: 0.8 mg/dL at the postoperative day 5). The edema of the forearm decreased day by day. The radial and ulnar pulses became palpable on the postoperative day 3. The fasciotomy wounds were closed with skin graft on the postoperative day 8. The patient was discharged from hospital with normofunctional left arm and hand on the postoperative day 10.

## 3. Discussion

Compartment syndrome is a clinical condition that is characterized by functional loss of muscle and nerve tissues and develops as a result of ischemia which can occur due to increased perfusion pressure within closed muscle fascia of the extremities. There are two types: acute and chronic compartment syndromes. Acute compartment syndrome develops when the intracompartmental pressure increases rapidly and it is often associated with trauma. Chronic compartment syndrome especially occurs due to excessive exercise or strain in the extremities [3].

There are many reasons, including bone fracture, crush injury in the muscle and soft tissues, intravenous (iv) or intra-arterial drug injections, brachial artery cannulation, brachial embolectomy, prolonged limb compression, IV thrombolytic treatment, vessel injury, burns, hemophilia, osteomyelitis, snake bites, insect bites (Crimean-Congo disease), postviral rhabdomyolysis, heavy lifting, aneurysms, during the electromyographic examination, and the use of a motorcycle for a long time [3–7].

This syndrome can be a complication encountered after vascular surgical procedures particularly applied to the lower extremities. In the literature, there are limited cases about reperfusion injury related to compartment syndrome of upper extremities [6, 7]. As seen in the reported cases, this syndrome can be limited to any part of upper extremities [7]. On the other hand this syndrome can comprise a whole forearm like our case.

The classical symptoms of compartment syndrome are ongoing severe pain and paresthesia, especially with passive stretching of the muscle. The classical findings are swelling, discoloration, and lose of pulse of the extremity. The deterioration of two-point discrimination is a sensitive finding and indicates the compartment syndrome [3]. The patient's symptoms and physical examination findings can be used for diagnosis. Compartment pressure measurement can be used to confirm the diagnosis and decide to administer fasciotomy [5]. In our case symptoms appeared in hours and the compartment syndrome was diagnosed by solely physical examination.

Allopurinol, steroids, superoxide dismutase, and mannitol can be helpful in suppressing and decreasing compartment syndrome [8]. Prophylactic fasciotomy also can be administered if there is a suspicion on developing compartment syndrome [4]. According to experimental studies, fasciotomy is recommended when intracompartmental pressure exceeds 30 to 45 mmHg [9]. The complications of urgent fasciotomy are associated mainly with its delay rather than the procedure itself. Fasciotomy wound infection is related to extent of tissue, duration of ischemia, and time gap of fasciotomy [8]. The functional deterioration of muscles occurs in 2–4 hours after ischemia and irreversible functional loss develops between 4th and 12th hours. Nerve injury starts after 30 minutes of ischemia and irreversible functional loss occurs after 12–24 hours. If fasciotomy can be carried out within 12 hours after onset of acute compartment syndrome, 68% of the patients return normal extremity function [10]. We carried out urgent fasciotomy at 4th hour after the beginning of complaints and did not encounter any complication associated with fasciotomy wound.

Anaerobic metabolism occurring during the ischemic phase creates an acidic environment. In the acidic environment myoglobin is released from ischemic muscle tissues. Acute tubular necrosis and acute renal failure occur as a result of myoglobin precipitation into the nephron [1]. Our patient was also faced with the same situation and was treated with hydration and diuresis with renal dose dopamine perfusion (3 mcg/kg/min). Renal functions returned to normal at the postoperative day 5.

## 4. Conclusion

Compartment syndrome may rarely occur after vascular surgical procedures applied to the upper extremities. This syndrome can be observed in diverse parts of upper extremity. Early diagnosis and immediate intervention are required in order to prevent contractures, neurological deficits, systemic effects, complete loss of the extremity, and death.
